# An integrative expression vector for *Actinosynnema pretiosum*

**DOI:** 10.1186/1472-6750-7-72

**Published:** 2007-10-24

**Authors:** Shan Goh, Andrea Camattari, Daniel Ng, Ruth Song, Kevin Madden, Janet Westpheling, Victor VT Wong

**Affiliations:** 1 Bioprocessing Technology Institute, 20 Biopolis Way, Centros #06-01, Singapore; 2 Microbia, Inc., 320 Bent Street, Cambridge, MA 02141, USA; 3 Genetics Department, University of Georgia, Athens, GA 30602, USA; 4 Department of Cell and Molecular Biology, Karolinska Institute, Berzelius väg 35, SE-171 77, Stockholm, Sweden

## Abstract

**Background:**

The Actinomycete *Actinosynnema pretiosum *ssp. *auranticum *has commercial importance due to its production of ansamitocin P-3 (AP-3), a potent antitumor agent. One way to increase AP-3 production would be to constitutively express selected genes so as to relieve bottlenecks in the biosynthetic pathway; however, an integrative expression vector for *A. pretiosum *is lacking. The aim of this study was to construct a vector for heterologous gene expression in *A. pretiosum*.

**Results:**

A series of integrative expression vectors have been made with the following features: the IS117 transposase from *Streptomyces coelicolor*, the constitutive ermE* promoter from *Saccharopolyspora erythraea*, different ribosome-binding site (RBS) sequences and *xylE *as a translational reporter. Positive *E. coli *clones and *A. pretiosum *transconjugants were assayed by catechol. pAP42, containing an *E. coli *consensus RBS, and pAP43, containing an *asm19 *RBS, gave strong and moderate gene expression, respectively. In addition, an operon construct capable of multi-gene expression was created. Plasmid integration sites in transconjugants were investigated and four different sites were observed. Although the most common integration site was within a putative ORF with sequence similarity to NADH-flavin reductase, AP-3 levels and cell growth of transconjugants were unaffected.

**Conclusion:**

A set of integrative vectors for constitutive gene expression in *A. pretiosum *has been constructed. Gene translation is easily determined by colorimetric assay on an agar plate. The vectors are suitable for studies relating to AP-3 biosynthesis as they do not affect AP-3 production.

## Background

*Actinosynnema pretiosum *is a commercially important organism due to its ability to produce ansamitocin P-3 (AP-3), a potent anti-tumor agent [1,2]. The cytotoxicity of ansamitocin has prompted its use as a toxic "warhead" in immuno-toxin conjugates [3]. Several of these conjugates are currently in late-phase clinical trials as therapeutic agents against solid tumors [4]. Thus, there is interest in generating strains of *A. pretiosum *that produce greater concentrations of AP-3 to meet increasing industrial demands, particularly as the yield from wild type *A. pretiosum *is low (~18 – 83 mg/l) [2,5]. Previously, a random mutagenesis approach [6] has been used to generate strains which produce 5- to 10-fold more AP-3 than the parental strain. Recently, deletion of a putative transcriptional repressor, *asm2*, has also been reported to increase AP-3 yield [7].

One method to improve the productivity of *A. pretiosum *would be to alter the regulation of ansamitocin biosynthesis through genetic manipulation of selected genes. The AP-3 biosynthetic genes, identified through comparisons with the *Amycolatopsis mediterranei *rifamycin biosynthetic gene clusters, and gene expression in *Streptomyces lividans *[8] and *S. coelicolor *[9], revealed the lack of a *rifH *homologue in *A. pretiosum *[9]. The *rifH *gene encodes an aminoDAHP synthase in *A. mediterranei *and is involved in the synthesis of aminoDAHP required for the AP-3 precursor, 3-amino-5-hydroxy-benzoic acid (AHBA). Addition of AHBA has been shown to increase AP-3 production [10]. Although DAHP synthase from the shikimate pathway in *A. pretiosum *may supply the AHBA pathway [9], it is not dedicated to aminoDAHP synthesis. Based on these reports, we hypothesized that a metabolic bottleneck in the synthesis of aminoDAHP was the limiting factor in AP-3 biosynthesis and sought to relieve this bottleneck through heterologous expression of *rifH*, thus providing an aminoDAHP synthase for *A. pretiosum*.

We report the construction of a series of novel expression vectors that allow stable integration of target genes into the *A. pretiosum *genome. The vectors have components from pSET152 [11], the IS117 transposable element [12] and the ermE* promoter [13], all of which have never previously been used in *A. pretiosum*. We have shown functionality of the vectors in *E. coli*, as the cloning host, and in *A. pretiosum*, as the transconjugant. We also validated plasmid constitutive expression, reporter function and integration preference, which did not alter host cell density or AP-3 levels. Finally, we demonstrate the vector's usefulness in heterologous expression of *rifH *in *A. pretiosum *and report its effects on AP-3 production.

## Results

### Conjugable and integrative pAP expression plasmids in *A. pretiosum*

**Figure 1:**
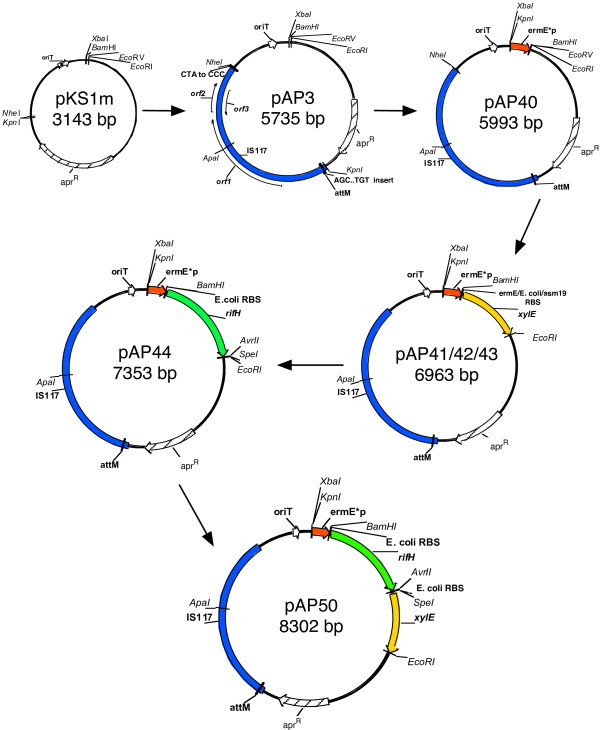
Construction of *A. pretiosum *vectors. Maps of plasmids with relevant features and single restriction sites are shown. Site directed mutagenesis of IS117 for a functional *attM *is indicated in pAP3. pAP41, pAP42 and pAP43 consist of *xylE *with RBS sequences from *S. erythraea*, *E. coli *and *A. pretiosum *genes, respectively, although only the *E. coli *RBS sequence is indicated. The *aac(3)IV *gene encoding apramycin resistance is represented by apr^R^.

IS117 is a well studied transposable element capable of integrating into several *Streptomyces *species [14,15] and into *Mycobacterium smegmatis *[16]. Plasmids containing IS117 had previously been derived from the mini-circle version [16,17]. In this study, IS117 was derived from a linearized and amplified copy in the *Streptomyces coelicolor *A3(2) chromosome and modified at the ends for integration into the chromosome of *A. pretiosum *(Figure 1). Plasmid containing IS117 with unmodified ends did not result in *A. pretiosum *transconjugants. By moving the *attM *(reverse complement) sequence, CTA, and the 15 bases upstream of it from the 3' end (downstream of orf2) to the 5' end (upstream of orf1), chromosomal integration at the *attM *site was restored. In this regard, consensus sequences of up to 19 nucleotides flanking *attM *have been identified in the genomes of *S. lividans *[14] and *M. smegmatis *[16], suggesting a role in alignment of the integration site. Transconjugants of pAP40 were apramycin resistant and circular plasmids were not detected in a chromosomal DNA preparation (not shown), which was expected, as the plasmid lacked a *Streptomyces *replicon.

**Figure 2:**
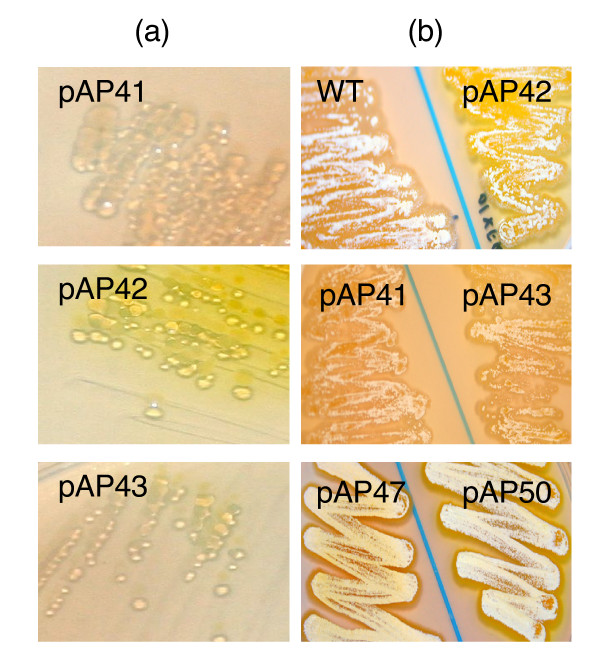
Catechol assay for *xylE *expression. (a) *E. coli *clones expressing *xylE *with an *E. coli *consensus RBS (pAP42), *ermE *RBS (pAP41) and *asm19 *RBS (pAP43). (b) *A. pretiosum *transconjugants of the same plasmids responded to catechol as in (a). Also shown are the operon construct (pAP50) and the fusion construct (pAP47).

The ermE*p is a strong promoter that has been used in *Streptomyces *for protein expression [18,19] and modulating secondary metabolites production [20-22], making it useful for this study. Three different RBS sequences were tested for efficiency: the unusual RBS sequence of *ermE *(pAP41) [13], an *E. coli *consensus RBS sequence (pAP42) [17], and a putative RBS sequence found upstream of the *A. pretiosum *gene, *asm19 *(pAP43) [9] (Figure 1). *E. coli *clones and transconjugants of pAP41, 42, and 43 were tested for metapyrocatachase activity, encoded by *xylE*, with catechol. Catechol positive colonies were observed strongly in *E. coli *clones of pAP42 and mildly in pAP43 clones while pAP41 clones were negative (Figure 2a). *A. pretiosum *transconjugants of pAP42 turned bright yellow, while transconjugants of pAP43 and pAP41 turned slightly yellow and did not change color, respectively (Figure 2b). This indicated the *E. coli *RBS sequence was the most robust, and that the ermE* promoter was functional in *A. pretiosum*. In addition, *xylE *was a suitable translational reporter in *A. pretiosum*.

To apply the reporter gene in an expression system, two versions of a translational reporter vector were constructed. Transconjugants of pAP47 (*xylE *and *rifH *fusion) did not result in catechol positive colonies while transconjugants of pAP50 (*xylE *and *rifH *operon; Figure 1) were catechol positive and their activities were comparable to transconjugants of pAP42 (Figure 2b).

### Constitutive expression of *rifH *in *A. pretiosum*

**Figure 3:**
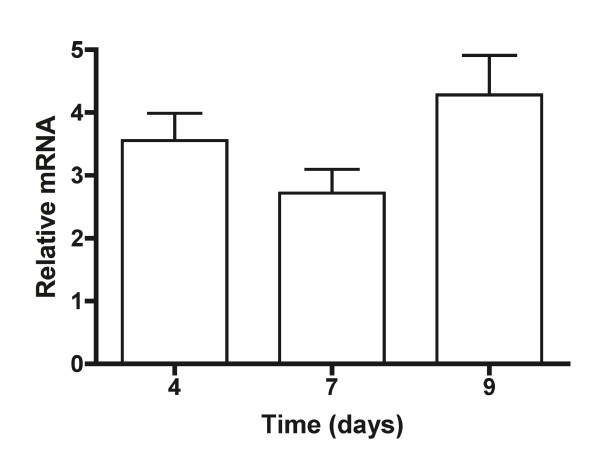
Constitutive expression of *rifH*. A clone of pAP50 grown in YMG shake-flasks in triplicate was harvested over 9 days and *rifH *mRNA abundance relative to day 2 was determined by qRT-PCR.

To determine whether the ermE* promoter enabled constitutive expression of *rifH *in *A. pretiosum*, a clone of pAP50 was grown in shake-flasks and cells were harvested over 9 days. Quantitation of *rifH *mRNA was normalized against 16S rRNA and calculated relative to the first harvest point (day 2). The amount of *rifH *mRNA on day 4, 7 and 9 did not change significantly (p = 0.16, by one-way ANOVA) (Figure 3).

### Integration sites, sequences and stability of pAP40, pAP42 and pAP50 in *A. pretiosum*

**Figure 4:**
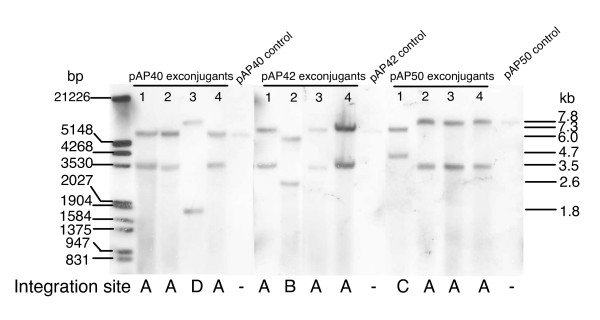
Integration sites in *A. pretiosum *transconjugants of pAP40, pAP42 and pAP50. Total DNA from apramycin resistant transconjugants were digested with *Apa*I and hybridized to a DIG-labeled IS117 probe. Unintegrated pAP40, pAP42 and pAP50 from *E. coli *were similarly digested and included as controls. Patterns of hybridized bands of each sample representing an integration site was arbitrarily labeled A, B, C and D. Site A consisted of an unchanged 3.5 kb band from the right arm of the integrated plasmid, and a variable left arm, dependent on the cloned gene of interest. Control plasmids have the same molecular weight as the left arm of its corresponding integrated plasmids because of an *Apa*I site on the genome, and not because of unintegrated plasmids in the total DNA extract. Transconjugants 1, 2, 3 and 4 of pAP40, pAP42 and pAP50 were subsequently tested for AP3 production.

**Figure 5:**
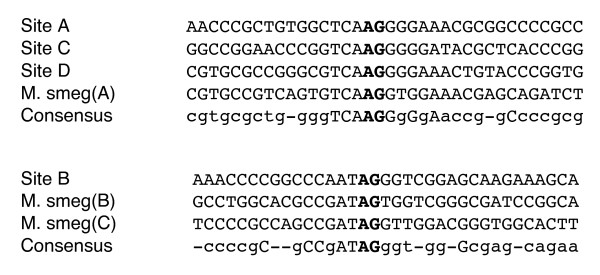
Alignment of IS117 integration sites in *A. pretiosum *and *M. smegmatis*. Bold letters indicate cross over point and capital letters indicate identical bases in all compared sequences.

It was important to determine the site of integration of the transconjugants to ensure the integrative vector was not affecting essential genes or genes involved in secondary metabolite production, and subsequently growth or AP-3 production. IS117 transposition integration has not been studied in *A. pretiosum*. Integration frequency was determined by Southern hybridization of genomic DNA from 12 transconjugants to an IS117 probe (Figure 4). No signal was observed from wild type *A. pretiosum *(not shown), while a single integration event at a specific site was observed in each transconjugant. Four specific integration sites (A, B, C and D) were found from sampling 12 transconjugants. The most common was A (9 of 12) while B, C and D each occurred once (Figure 4). The sequences of integrated plasmids and chromosomal junctions were determined by plasmid rescue, which allowed the design of site A, B, C and D specific PCR primers (Table 1) and amplification of the chromosomal integration sites. Comparison of the IS117 integration sites in *A. pretiosum *to those in *M. smegmatis *revealed consensus sequences for *A. pretiosum *sites A, C and D and *M. smegmatis *site A, and *A. pretiosum *site B and *M. smegmatis *sites B and C (Figure 5). Sequence analysis revealed that *A. pretiosum *site A was within an ORF with homology to a putative NADH-flavin reductase of *Nocardia farcinica *IFM 10152 (gi 54024270, E-value 1e-13, similarity in 70 of 192 aa). Sites B, C and D were in intergenic regions, upstream of a putative integral membrane protein of *Thermobifida fusca *YX, downstream of a glutamyl-tRNA synthetase of *Nocardia farcinica *IFM 10152, and upstream of a putative ATP-dependent RNA helicase of *N. farcinica *IFM 10152, respectively. The pAP plasmids did not cause target site duplication upon integration. PCR amplification of chromosomal and plasmid junctions was used to detect tandem insertions, which would result in amplicons of 272 bp and > 6 kb, or deletions, which would not result in an amplicon. Tandem insertions or deletions were not detected in the transconjugants by Southern hybridization or PCR (not shown).

**Table 1:** Primers used in this study.

Primer	Sequence (5' – 3')
Vector construction	
IS117-KpnI	TGACTGGTACCGGAGTCGGGGATGTTCTTGTTC*
IS117-NheI	TGACTGCTAGCGTACCGGTGCCCCGATAGAC
ermE-XbaI/KpnI	AAAATCTAGAGGTACCAGCCCGACCCGAGCA
ermE-BamHI/EcoRV	AAAAGATATCGGATCCACCAACCGGCACGA
xylE-F4Bam	CGCGGATCCAGCGATGAACAAAGGTGTAATGCGACC
xylE-FEBam	CGCGGATCCAAGCTAACGTAAGGAGGAAAAACATATGAACAAAGGTGTAATGCGACCGGGCCAT
xylE-FABam	CGAGGATCCTCCCCACAGACAGGGAGCCCAGCATATGAACAAAGGTGTAATGCGACCGGGCCAT
xylE-REcoRI	GCGGAATTCTCAGGTCAGCACGGTC
rifH-FNdeI	CGCATACATATGGTGAAGCGGCAGCCGGACTTCG
rifH-RASE	CGCGAATTCACTAGTCCTAGGCTAGCGCAGCATCTTCGCGATCG
xylE-FSpeI	AATACTAGTATGAACAAAGGTGTAATGCGACCGGGCCAT
xylE-FRBSSpeI	CGCAATACTAGTAAGCTAACGTAAGGAGGAAAAACATATGAACAAAGGTGTAATGCGACCGGGCCAT
qPCR	
rifH-668F	AGATCTACGTCAGCCACGAAATG
rifH-788R	TCGCCGATCCACAGGAA
16S-F	CAGAAGAAGCACCGGCTAAC
16S-R	TTAAGCCCCAAGTTTTCACG
Southern hybridization	
IS117-F	CTGAACTCACCGCGACGTATCG
IS117-R	GCTAGCGTACCGGTGCCCCG
Plasmid rescue and sequencing	
intseq1	CCGTTTGGCCTCCGACTAAC
intseq2	GCTCTATCGGGAGGCCTCAC
attM5'1	GGAGCAGACGCTCGTCCGGG
siteA-F	GTCGTGGTCACCAGGCGGAC
siteA-R	ACCGCCTTCGTCCGTGCCAG
siteB-F	GATCTGTGTTCGCGGAGAGC
siteB-R	ACAAGGGCGTCAGCGAGGAA
siteC-F	GGTGTCCTCGATGCGGAAGA
siteC-R	AAGTCCCCGTGCTTCTCCAG
siteD-F	GACCCCCTCCGCGAACTGGT
siteD-R	GGCCTGCGAGGTTTGTGCGC

*Restriction sites are underlined

Stability of plasmids integrated into sites A, B, C and D of *A. pretiosum *was determined. After 50 duplications under non-selective conditions, cultures representing each of the integration sites had less than 1% plasmid loss.

### AP-3 production of transconjugants

**Figure 6:**
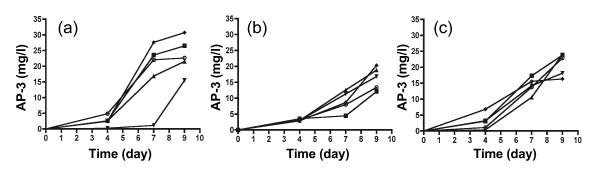
AP-3 production of *A. pretiosum *transconjugants in YMG in shake flasks. AP-3 concentration was measured over 9 days. (a) Transconjugants 1 (■), 2 (▲), 3 (▼) and 4 (◆) of pAP40 compared to WT (○). (b) Transconjugant 1 (■), 2 (▲), 3 (▼) and 4 (◆) of pAP42 expressing *xylE*, compared to WT (○). (c) Transconjugant 1 (■), 2 (▲), 3 (▼) and 4 (◆) of pAP50 expressing *rifH*, compared to WT (○).

Since the most common site of plasmid integration was within a putative ORF, the effect of integration on AP-3 production was of interest. Mean AP-3 yields from WT in YMG obtained on ten different occasions were 13.3 ± 5.6 mg/l for day 7 and 15.3 ± 5.1 mg/l for day 9. Transconjugants 1 to 4 of pAP40, pAP42 and pAP50 having either site A, B, C or D plasmid integrations were assayed for AP-3 production in YMG. For pAP40 transconjugants, site A mutants did not affect AP-3 yield over 9 days, but the site D mutant had lower AP-3 yields, particularly on day 7 (Figure 6a). All pAP42 and pAP50 transconjugants expressing *xylE *and *rifH*, respectively, had similar AP-3 yield to the WT at the end of the fermentation period (Figure 6b &6c). Packed cell volume of transconjugants, representing cell growth, was also measured for each AP-3 assay and found to be equivalent to that of WT (not shown).

## Discussion

An important factor in expression vectors is a suitable RBS; failure of pAP41 for *xylE *expression in *E. coli *was likely due to host ribosome and RBS incompatibility. The vector with an *E. coli *consensus RBS sequence (pAP42) resulted in the strongest expression of *xylE*, while the putative RBS sequence of *A. pretiosum asm19 *(pAP43) resulted in weaker expression. Hence, while pAP42 is useful for strong constitutive expression of target genes, pAP43 may be used if moderate expression is required. To confirm translation of the gene of interest, two versions of a translational reporter vector were made. The pAP47 *rifH-xylE *fusion construct (not shown) did not produce a functional metapyrocatachase, perhaps due to steric hindrance of the fused protein or the lack of appropriate translational signals for *xylE*, while the pAP50 *rifH-xylE *operon construct with an RBS sequence dedicated to the reporter gene worked well.

For the purposes of this study, it was important that the integrative plasmid did not affect host AP-3 production or bacterial growth. Hence, plasmid integration sites and their effect on AP-3 levels were determined. Plasmid integration into site A was most common, while sites B, C and D occurred once in each set of transconjugants containing either pAP40, pAP42 or pAP50. Since only four transconjugants of each set were screened, it is unlikely these secondary sites are plasmid-dependent. Comparison of *A. pretiosum *integration sites with those of *M. smegmatis *and hence *S. lividans *revealed GC-rich consensus recognition sequences flanking the *attM *that are broadly categorized into two groups, consisting of either AG or TAG as the cross-over sequence.

Integration of a plasmid into site A may have inactivated a putative ORF region, however unchanged AP-3 levels and packed cell volume in mutants indicate that a preference for site A did not interfere with AP-3 biosynthesis or cell growth. Similarly, integration into secondary sites of B, C and D did not markedly affect AP-3 production after 9 days. Although *rifH *mutants also had unchanged AP-3 levels, suggesting the presence of aminoDAHP synthase does not have any effect on AP-3 biosynthesis, more work needs to be done to assess the activity of RifH in transconjugants, and to optimize substrates in the growth media, to draw any final conclusions on the effect of RifH modulation on AP-3.

## Conclusion

We have constructed a series of useful genetic tools for the industrially valuable bacterium *A. pretiosum*. Stable maintenance, translational reporter function in *E. coli *and *A. pretiosum*, strong and moderate constitutive gene expression and lack of any effect on AP-3 production and bacterial growth are all desirable features of the pAP42/43/50 vectors.

## Methods

### Bacterial strains, plasmids and growth conditions

Media was prepared from DIFCO stocks and antibiotics were purchased from Sigma, unless otherwise stated. *A. pretiosum *and *E. coli *strains used in this study are listed in Table 2. *A. pretiosum *spore stocks were prepared as previously described [23], and working stocks were either subcultured from spore stocks or a single colony grown on YMG agar (4 g/l yeast extract, 10 g/l malt extract, 4 g/l glucose, 20 g/l agar, pH 7.2) or MYM agar (4 g/l yeast extract, 4 g/l maltose, 10 g/l malt extract, 20 g/l agar) at 26°C for 2–3 days, and inoculated in YMG or VM (5 g/l meat extract, 5/L g peptone, 5 g/l yeast extract, 2.5 g/l enzyme hydrolysate of casein, 20 g/l glucose, 1.5 g/l NaCl) [24]. *A. pretiosum *mutants containing the integrative vector were grown in media supplemented with apramycin (50 μg/ml). *E. coli *JM109, XL1 Blue and XL10 Gold transformants were cultured in LB medium at 37°C supplemented with apramycin (50 μg/ml). ET12567/pUZ8002 transformants was maintained in chloramphenicol (25 μg/ml), kanamycin (25 μg/ml) and apramycin (50 μg/ml).

**Table 2:** Bacterial strains and plasmids used in this study

Strain or plasmid	Relevant characteristic	Purpose	Reference/Source
*Amycolatopsis mediterranei*	Rifamycin producer	Amplification of *rifH*	ATCC
*S. coelicolor A(3)2*	IS117 transposable elements	Amplification of IS117	ATCC
*A. pretiosum *31565	AHBA biosynthesis gene cluster	Amplification of *asm19 *RBS, conjugation	ATCC
*E. coli*			
ET12567/pUZ8002	Methylation-deficient host with non-transmissible helper plasmid	Conjugation host	[25]
Plasmids			
pKS1	apr^R^, ori	Vector backbone	This study
pXE3	*xylE*	Amplification of *xylE*	[25]
pAP40	ermE*p, IS117, *aac(3)IV*	Assessment of IS117	This study
pAP41	Derived from pAP40, *ermE *RBS, *xylE*	Assessment of RBS	This study
pAP42	Derived from pAP40, *E. coli *RBS, *xylE*	Assessment of RBS	This study
pAP43	Derived from pAP40, putative *asm19 *RBS, *xylE*	Assessment of RBS	This study
pAP44	Derived from pAP42, *E. coli *RBS, *rifH*	Construction of translational reporter	This study
pAP47	Derived from pAP44, *E. coli *RBS, *rifH*-*xylE *fusion	Assessment of translational reporter	This study
pAP50	Derived from pAP44, *E. coli *RBS, *rifH*-*xylE *operon construct	Assessment of translational reporter	This study

### Construction of pAP plasmids

Construction of the pAP plasmids are summarized in Figure 1. The plasmid pKS1, derived from pSET152 by digestion with *Sph*I and *Hind*III and treated with Klenow before ligation, consisted of the apramycin resistance gene (*aac(3)IV*) and oriT but not the ϕC31 integrase and attachment site. IS117 was amplified from an integrated linear copy in the *S. coelicolor *A3(2) genomic DNA using primers IS117-KpnI and IS117-NheI (Table 1). The IS117 PCR product, consisting of orf1, 2 and 3, was digested with *Kpn*I and *Nhe*I and cloned into similar ends in a modified pKS1 with a *Kpn*I site (pKS1m). IS117 was modified to obtain a functional *attM *firstly by mutation of CTA to CCC downstream of orf2, and secondly by inserting AGCCCCCTGAGATGT upstream of CTA at the 5' end of orf1 by site-directed mutagenesis, resulting in pAP3. The ermE* promoter was amplified from pIJ4090 [23] using primers ermE-XbaI/KpnI and ermE-BamHI/EcoRV (Table 1) digested with *Xba*I and *EcoR*V and cloned into pAP3 at similar sites to create pAP40.

The reporter gene *xylE *with different types of RBS was amplified from pXE3 [25] with forward primers specifying an RBS from either the ermE* promoter [13] (xylE-F4Bam), *E. coli *genes [17] (xylE-FEBam) or *asm19 *(gi 21449342) of *A. pretiosum*.(xylE-FABam), and a reverse primer (xylE-REcoRI). The different *xylE *PCR products were digested with *BamH*I and *EcoR*I and cloned into pAP40 with similar ends to produce pAP41, 42 and 43 having the *ermE *RBS, *E. coli *consensus RBS and *asm 19 *RBS, respectively, upstream of *xylE*. Clones of pAP41, 42 and 43 were used in catechol assays to determine functionality of RBS.

*rifH *(gi 41581793) was amplified from the *A. mediterranei *genome with primers rifH-FNdeI and rifH-RASE. The *xylE *gene was excised from pAP42 at *Nde*I and *EcoR*I to allow cloning of the *rifH *PCR product, which was digested with the same restriction enzymes. This resulted in plasmid pAP44 containing an *E. coli *RBS upstream of *rifH*. Translation of *rifH *was determined by creating a *xylE *fusion and an operon construct of *rifH *and *xylE*. For the fusion recombinant, *xylE *was amplified from pXE3 with xylE-FSpeI and xylE-REcoRI. The PCR product was digested with *Spe*I and *EcoR*I and cloned into pAP44 with similar ends. The *rifH *stop codon was then removed to enable translational read through to *xylE *(pAP47, not shown). For the operon construct, *xylE *was amplified with a forward primer containing an *E. coli *RBS (xylE-FRBSSpeI) and xylE-REcoRI. The PCR product was digested with *Spe*I and *EcoR*I and cloned into pAP44 with similar ends to result in pAP50. Clones of pAP50 were examined for constitutive expression of *rifH *by qRT-PCR and AP-3 production in YMG.

### Transformation and conjugation

Transformation of *E. coli *was performed by electroporation using a Gene Pulser (Bio-Rad) as recommended by the manufacturer. Conjugation between ET12567/pUZ8002 and *A. pretiosum *was as described previously [23]. The integrative vectors were transformed into ET12567/pUZ8002 and selected with apramycin on LB agar plates. Selected transformant colonies were grown in LB medium supplemented with kanamycin (25 μg/ml), chloramphenicol (25 μg/ml) and apramycin (50 μg/ml) at 37°C for 20 hours. A 1/50 dilution of the *E. coli *was made and grown for 4–5 hours at 37°C to an OD_600 _of 0.4–0.6. The cells were harvested, washed twice with equal volumes of LB and resuspended in 0.1× original volume. *A. pretiosum *spores (~10^8^) in 2 × YT were heat-shocked at 50°C for 10 min, then mixed with the *E. coli *suspension by swirling. The mixture was plated on mannitol soy agar (MS)+10 mM MgCl_2 _and incubated at 37°C for 16 h. The plates were overlaid with 4 ml nutrient soft agar (0.5% w/v) supplemented with nalidixic acid (120 μg/ml) and apramycin (60 μg/ml), and incubated at 26°C for 5–7 days. Transconjugants were picked and transferred to fresh YMG plates supplemented with apramycin (50 μg/ml) and nalidixic acid (25 μg/ml). Spore stocks were subsequently prepared from the single colonies.

### Plasmid stability of transconjugants

*A. pretiosum *transconjugants of pAP40, pAP42 and pAP50, representing plasmids integrated at sites A, B, C and D were tested for plasmid stability [26]. A single colony from MYM with apramycin (50 μg/ml) was inoculated into VM (80 ml) and grown at 26°C at 180 rpm for three days. An aliquot of the culture was appropriately diluted and plated onto MYM, while the remaining culture was grown in VM at 26°C with shaking. Colonies (200 cfu) from the day three MYM plate were transferred to MYM with apramycin (50 μg/ml) to obtain a ratio of resistant cells to total cells. After propagating the transconjugant culture for approximately 50 duplications in VM, the ratio of apramycin resistant cells to total cells was determined as before, and plasmid loss was also calculated.

### Plasmid rescue

To determine the site of vector integration in transconjugants, plasmid rescue was carried out with *Asc*I, which is a non-cutter for the pAP plasmids. Genomic DNA (1 μg) with integrated plasmid was digested with *Asc*I and ligated with T4 DNA ligase (NEB) at 16°C for overnight. The ligation reaction was electroporated with *E. coli *JM109 cells and transformants were selected on LB agar with apramycin (50 μg/ml). Plasmid DNA was extracted from randomly selected transformants and sequenced with primers intseq1 and attM5'1 for the right hand plasmid-chromosome junction, and intseq2 for the left hand junction (Table 1). Primers specific for the bacterial chromosome to the left and right of each integration site (siteA-F, siteA-R; siteB-F, siteB-R; siteC-F, siteC-R; siteD-F, siteD-R; Table 1) were used to determine sequences flanking the integrated plasmid, as well as the integration site of the wild type genome. Sequence analyses were carried out by BLASTN and BLASTX.

### Nucleic acid extractions

Plasmid DNA extractions were carried out using Qiaprep Spin Miniprep kit (Qiagen) according to manufacturer's specifications, while genomic DNA extractions were carried out as previously described [23]. A three day culture of *A. pretiosum *in 30 ml VM was washed twice in 10.3% (w/v) sucrose, treated with lysozyme (1 mg/ml), proteinase K (560 μg/ml), SDS (1% v/v) and RNase A (100 μg/ml). The lysate was washed three times with equal volumes of phenol: chloroform: isoamyl alcohol (Sigma) and once in an equal volume of chloroform. DNA was precipitated in 2 M NaCl and 0.6 × isopropanol, washed twice in 70% (v/v) ethanol, air-dried and dissolved in 10 mM Tris-Cl (pH 8).

Total RNA extraction of *A. pretiosum *grown in YMG broth harvested at days 2, 3, 4, 7, 8 and 9 was carried out using the RiboPure™-Bacteria Kit (Ambion), with the following modifications. Cultures (7–10 ml) were pelleted and stored at -80°C until extracted. Cell pellets were resuspended in 0.5 × original volume of DEPC water (0.1%, v/v), freeze-thawed five times in liquid nitrogen and at 55°C, then treated with lysozyme (3.5 mg/ml) at 37°C for 20 min. Cells were pelleted, resuspended in 350 μl of RNAwiz, vortexed with zirconia beads for 15 mins before continuing with the manufacturer's protocol. RNA was treated with DNase I, electrophoresed in a 1% TAE agarose gel and quantified spectrophotometrically.

### Quantitative RT-PCR (qRT-PCR) and sequencing

RNA (1 μg) from three biological replicates was converted to cDNA using random hexamers and the iScript cDNA Synthesis kit (Bio-Rad) in a 20 μl reaction. Subsequently, 5 μl of cDNA was used for *rifH *amplification. For 16S rRNA amplification, cDNA was diluted 10^6^-fold and 5 μl was used for quantitative PCR (qPCR). Amplification was carried out in an ABI Prism 7000 sequence detector where each amplification reaction contained 12.5 μl of iTaq SYBR Green Supermix with Rox (Bio-Rad) and 300 nM each of forward and reverse primers in a 25 μl reaction. RNA from three biological replicates was used and qPCR of each cDNA sample was carried out in triplicate. Primers were designed by Primer Express (Applied Biosystems) and are as follows: rifH-688F and rifH-788R for the target gene *rifH *and 16S-F and 16S-R for the reference gene 16S rRNA (Table 1). The cycling conditions were 3 min at 95°C, followed by 45 cycles of 15 s at 95°C and 45 s at 58°C. Efficiencies of both primer pairs were found to be similar, hence, data was analyzed by the 2^-ΔΔCT ^method [27]. Sequencing was carried out in an ABI Prism 3100 Genetic Analyzer with Big-Dye chemistry.

### Southern hybridization

The frequency and site of vector integration in *A. pretiosum *transconjugants were determined by Southern hybridization. Genomic DNA (4–6 μg) digested with *Apa*I was hybridized with DIG-labeled IS117 probe (25 ng/ml) using the DIG-labeling and detection starter kit (Roche) [28]. DIG-labeled probe was derived from 1 μg of IS117 amplified from pAP40 with primers IS117-F and IS117-R (Table 1). Hybridization was carried out at 40°C and stringency washes were performed in 0.1 × SSC at 65°C.

### Catechol assay

Catechol assay was carried out as previously described with modifications [25]. Streaks were made from single colonies of the various *E. coli *transformants onto LB plates or *A. pretiosum *transconjugants onto YMG plates containing 50 μg/ml apramycin. After one (for *E. coli*) or two (for *A. pretiosum*) days, 0.5 M aqueous catechol (Sigma) was sprayed onto the surface of the plates containing the colonies and incubated in the dark at 28°C for 40 min. Positive *xylE *expression was seen as a yellow halo around bacterial streaks.

### Extraction and quantification of AP-3

A single colony of either mutant or wild type *A. pretiosum *from a working stock plate was subcultured into 10 ml of YMG with or without apramycin, respectively. The culture was incubated for 9 days at 26°C with shaking, and aliquots were removed on days 4, 7 and 9 for AP-3 measurement as follows:1 ml was centrifuged at 3270 × *g *for 15 min and 400 μl of supernatant was mixed with 7.6 ml of ethyl acetate (Merck) for 1 min by vortexing and centrifuged as above at 4°C, while the cell pellet (packed cell volume) was weighed and noted. The organic phase of the supernatant mixture was transferred to a fresh tube, evaporated in a nitrogen sample concentrator (Techne) and the desiccated material was resuspended in 200 μl of a 60%/40% (v/v) solution of solvent A (0.1% formic acid (Merck) in high purity water) and solvent B (0.1% formic acid in methanol). The sample was eluted at 0.8 ml/min in a Shimadzu chromatographer with a Hypurity C18 column (Thermo) with the following gradient pattern: 45% solvent B for 10 min, 45–70% solvent B for 20 min, 80% solvent B for 5 min and 45% solvent B for 20 min. The concentration of AP-3 in the sample was determined by peak comparison with an AP-3 standard (Calbiochem) of known concentration.

### Statistical analysis

Statistical significance was determined by one-way analysis of variance (ANOVA) test performed using Microsoft Excel. A p-value < 0.05 was considered significant.

## Competing interests

The author(s) declares that there are no competing interests.

## Authors' contributions

SG carried out vector construction and other genetic manipulations, the ANOVA test, participated in study design and drafted the manuscript. AC carried out plasmid rescue, determined plasmid stability and participated in AP-3 assays. DN carried out qRT-PCR, statistical analysis and participated in AP-3 assays. RS carried out Southern hybridization and RNA extraction. KM and JW participated in study design and helped draft the manuscript. VW conceived the study, carried out vector construction and AP-3 assays and helped with study design and drafting of the manuscript. All authors read and approved the final manuscript.
